# The New Era of Total Neoadjuvant FLOT Therapy for Locally Advanced, Resectable Gastric Cancer: A Propensity‐Matched Comparison With Standard Perioperative Therapy

**DOI:** 10.1002/jso.27934

**Published:** 2024-10-13

**Authors:** Ahmet Rencuzogullari, Salih Nafiz Karahan, Fatih Selcukbiricik, Sahin Lacin, Orhun Cig Taskin, Burcu Saka, Duygu Karahacioglu, Bengi Gurses, Emre Ozoran, Derya Salim Uymaz, Ibrahim Halil Ozata, Sezer Saglam, Dursun Bugra, Emre Balik

**Affiliations:** ^1^ Department of General Surgery, School of Medicine Koç University Istanbul Turkey; ^2^ Department of Medical Oncology, School of Medicine Koç University Istanbul Turkey; ^3^ Department of Pathology, School of Medicine Koç University Istanbul Turkey; ^4^ Department of Radiology, School of Medicine Koç University Istanbul Turkey; ^5^ Department of Medical Oncology Demiroglu Bilim University Istanbul Turkey; ^6^ Department of General Surgery American Hospital Istanbul Turkey

**Keywords:** FLOT therapy, gastric cancer, perioperative therapy, total neoadjuvant therapy

## Abstract

**Background:**

The FLOT 4‐AIO trial established the docetaxel‐based regimen's superiority over epirubicin‐based triplet therapy in terms of survival rates and acceptable toxicity for locally advanced resectable gastric (LARGC). Yet, fewer than half of the patients achieved completion of eight prescribed FLOT cycles. We proposed that administering all FLOT cycles in the form of total neoadjuvant therapy may improve completion rates and downstaging. This study contrasted total neoadjuvant therapy (FLOT x8) with standard neoadjuvant therapy (FLOT 4+4) for patients LARGC adenocarcinoma who underwent curative resection with routine D2 lymphadenectomy, focusing on histopathological outcomes, toxicity, and survival outcomes.

**Methods:**

We reviewed patients with histologically confirmed advanced clinical stage cT2 or higher, nodal positive stage (cN+), or both, with resectable gastric tumors and no distant metastases (January 2017 to July 2023). We divided patients into two groups, FLOT 4+4 and FLOT x8; FLOT 4+4 patients underwent four preoperative and four postoperative bi‐weekly cycles of docetaxel, oxaliplatin, leucovorin, and fluorouracil, while FLOT x8 patients received all eight cycles preoperatively after a gradual practice change starting from January 2020. Propensity score matching adjusted for age, clinical stage, tumor location, and histology.

**Results:**

Of the 77 patients in the FLOT x8 group, 37 were propensity‐matched to an equal number in the FLOT 4+4 group. Demographics, duration of surgery, and hospital stay showed no significant differences between the groups. The FLOT x8 group exhibited a significantly higher all‐cycle completion rate at 89.1% compared to FLOT 4+4's 67.6% (*p* < 0.01). Both groups demonstrated comparable hematological and non‐hematological toxicity rates, Clavien−Dindo ≥ 3 complications, and CAP tumor regression grades. The mean number of harvested lymph nodes was 42.5 and 41.2 in the FLOT 4+4 and FLOT x8 groups, respectively. Similar rates of disease‐free survival and overall survival were noted in both groups, despite a trend toward a higher pathological complete response rate, albeit not statistically significant (8.1% vs. 18.9%, *p* = 0.29), in the FLOT x8 group at a median follow‐up of 36 months.

**Conclusion:**

Total neoadjuvant therapy with the FLOT x8 protocol corresponds to higher treatment completion rates, a safety profile similar to standard perioperative therapy, and a twofold increase in complete pathological response. Further research on long‐term oncological outcomes is needed to confirm the effectiveness of total neoadjuvant therapy.

## Summary

- This study evaluates total neoadjuvant therapy (TNT) using eight cycles of the FLOT regimen (FLOT x8) for locally advanced, resectable gastric cancer patients who underwent gastrectomy with standardized D2 lymphadenectomy, comparing it with the standard perioperative FLOT therapy (FLOT 4+4).
- TNT with FL OT x8 showed a significantly higher all‐cycle completion rate (89.1%) compared to the perioperative approach (67.6%), suggesting better tolerance and feasibility before surgery.
- Both groups had similar perioperative outcomes (duration of surgery, length of hospital stay, i.e.,) and postoperative complications and hematologic and non‐hematologic toxicity rates, indicating the safety of the TNT approach.
- While similar rates of disease‐free survival and overall survival were noted in both groups, the pathological complete response rate in FL OT x8 nearly doubled.
- Further research on long‐term outcomes of TNT with FLOT x8 followed by gastrectomy with standardized D2 lymphadenectomy is essential.

## 1 Introduction

The curative treatment for gastric cancers is only possible in early‐stage (T1) disease, with a dramatic decrease in survival observed in advanced‐stage disease. The 5‐year survival rate for T3/4 disease is 20% [1]. Strategies established through multidisciplinary assessment have become crucial in improving survival rates. Compared to surgery alone, the MAGIC study demonstrated a survival advantage with perioperative chemotherapy for gastric and esophagogastric junction adenocarcinomas managed with a D2 dissection rate of about 40% [2]. A regimen comprising epirubicin, cisplatin, and fluorouracil or capecitabine (ECF/ECX), administered in three cycles before and after surgery, resulted in a 36% 5‐year survival advantage compared to 23% with surgery alone. The perioperative FLOT regimen, which has become the current standard for locally advanced resectable gastric cancer (LARGC), has superseded the ECF/ECX regimen due to allowing higher resection rates and associated survival benefits. The landmark research, the FLOT 4‐AIO study, reported a 50‐month survival superiority with four cycles each of preoperative and postoperative chemotherapy (FLOT: docetaxel 50 mg/m², intravenous oxaliplatin 85 mg/m², intravenous leucovorin 200 mg/m², and fluorouracil 2600 mg/m²), compared to 35 months survival of ECF/ECX regimen [3]. However, both MAGIC and FLOT 4‐AIO studies reported notably low completion rates for planned therapy with rates of around 40% [2, 3].

The difficulty in completing the full course of chemotherapy is largely attributed to the postoperative setting. In the MAGIC study, 86% of patients assigned to perioperative chemotherapy completed the preoperative chemotherapy, while 41.6% completed all six cycles [2]. Similarly, in the FLOT 4‐AIO study, while 91% of patients in the ECF/ECX arm completed their planned preoperative chemotherapy, only 37% managed to finish the postoperative cycles. In the FLOT arm, 90% of patients completed their preoperative chemotherapy cycles, but only 46% were able to complete the postoperative treatment [3]. These findings underscore the postoperative period as a key barrier to optimal treatment outcomes, justifying the exploration of alternative strategies, such as total neoadjuvant therapy (TNT), which concentrates the entire chemotherapy regimen in the preoperative phase.

Recognizing noncompliance with the postoperative phase of the treatment, in early 2020, we transitioned from using the standard perioperative FLOT regimen (FLOT 4+4) to adopting TNT, which involves administering all eight cycles of FLOT therapy preoperatively (FLOT x8), for LARGC. This approach was introduced in a phased manner as part of our institutional multidisciplinary consensus to optimize further the current recommendations of perioperative FLOT therapy—outlined by the NCCN guidelines for gastric cancer—which splits the regimen into four cycles before and four cycles after surgery [4]. The rationale behind this practice change was based on the premise that implementing total neoadjuvant FLOT therapy would enhance the rate of completion of chemotherapy cycles and subsequently lead to improved histopathological outcomes. This provided the opportunity for a retrospective comparison between perioperative and TNT, both with FLOT regimes. The primary aim of this study is to compare TNT with FLOT x8 with standard perioperative therapy (FLOT 4+4) for LARGC, undergoing D2 lymphadenectomy as the standard of care, in terms of cycle completion rate. Secondary endpoints included histopathological response, toxicity, and survival rates.

## 2 Methods

### 2.1 Patient Selection and Group Descriptions

This two‐center, single‐institution retrospective study conducted an intention‐to‐treat analysis of a cohort of consecutive patients who received peri‐ or total neoadjuvant FLOT therapy from January 2018 through June 2023 at Koç University Hospital and American Hospital, both part of the Koç Healthcare Group. The multidisciplinary tumor board (MTB) in the Koç healthcare group regularly reviews all gastric cancer patients, and individualized treatment decisions are made in conjunction. Patients > 18 years old who underwent curative surgery for biopsy‐proven gastric cancer were included if they were at a clinical stage of ≥ T2 or had a node‐positive status. Patients were excluded if they had metastatic disease, a known history of previous malignancy, a histopathologic diagnosis other than adenocarcinoma, were lost to follow‐up, or underwent palliative or emergency surgery.

Patients were categorized into two groups based on the number and timing of FLOT chemotherapy cycles they received. The FLOT 4+4 group consisted of patients who received a combination of four preoperative and four postoperative bi‐weekly cycles of docetaxel, oxaliplatin, leucovorin, and fluorouracil. In contrast, the FLOT x8 group consisted of patients who received all eight cycles of chemotherapy in the preoperative phase, with no adjuvant therapy administered postoperatively.

**Figure 1:**
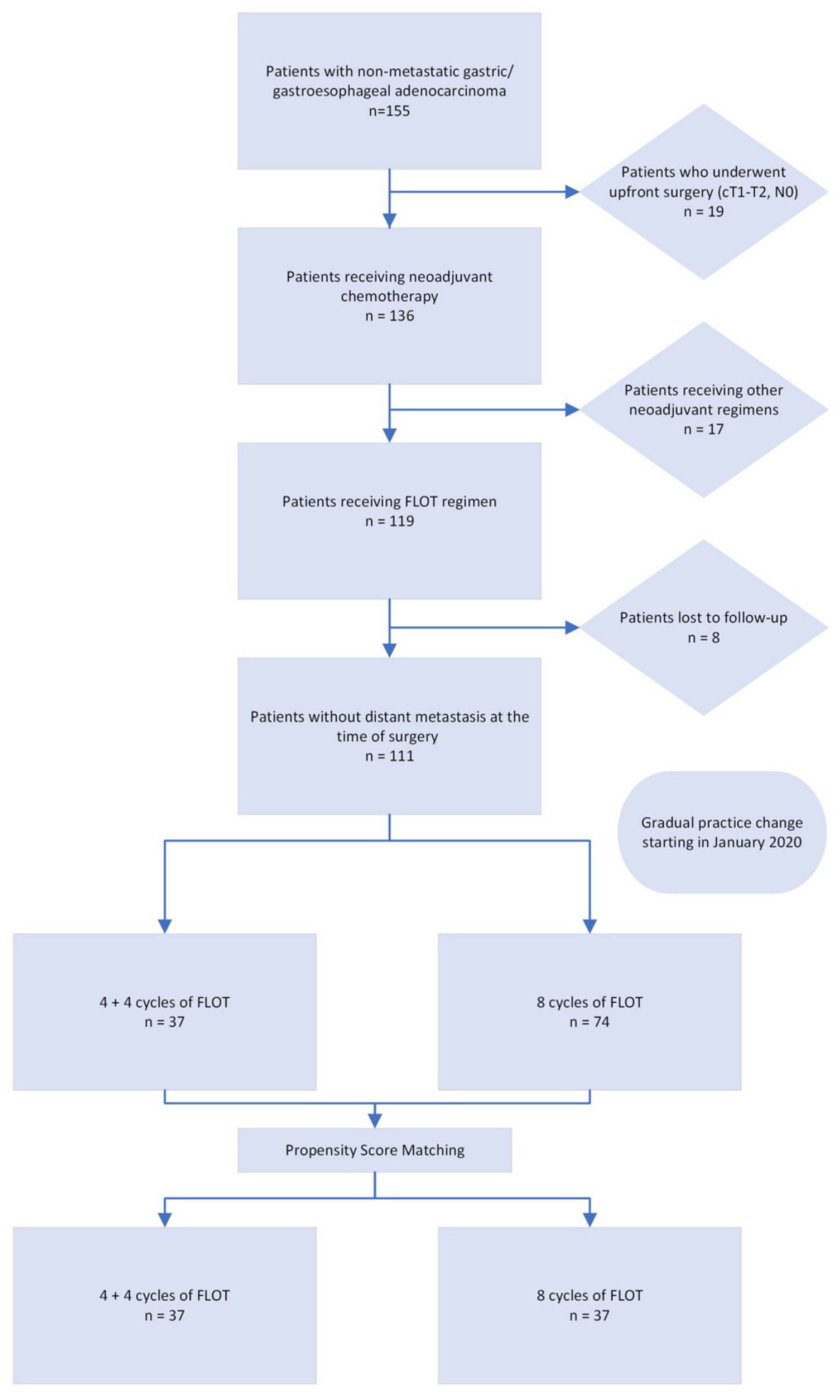
Flowchart of the study design and patient enrollment.

A study flowchart is depicted in Figure 1. The protocol received approval from the Koç University Institutional Review Board (IRB) (2022.439.IRB1.165) and was conducted in accordance with the principles outlined in the Declaration of Helsinki. Individual consent for this retrospective analysis was waived.

### 2.2 Staging and Evaluation

The clinical stage was determined using the latest version of the AJCC staging system. Evaluations included physical examination, esophagogastroduodenoscopy, endoscopic ultrasound for early gastric cancer (T1−2), and computed tomography (CT) scans of the chest, abdomen, and pelvis. “Node‐positive” status was defined based on suspicious lymph nodes observed on CT or endoscopic ultrasound [5] with criteria for nodal involvement including a small axis diameter of ≥ 6 mm for perigastric LNs and ≥ 8 mm for extra‐perigastric LNs, along with morphological characteristics such as round shape, heterogeneous or intense enhancement, and the presence of clusters of three or more nodes [6].

Abdominopelvic CT was performed after ingestion of 600−1000 mL of water as a negative enteral contrast agent and administration of intravenous contrast, and the images were acquired at the venous phase. All CT examinations were performed after 6 h of fasting. The same protocol was applied both at the time of initial staging and response evaluation. Given the limited performance of CT in T staging of gastric cancer, there was no attempt to differentiate between T1 and T2, and T3 and T4 in the context of this study [7]. Whenever the tumor was at least T3 (subserosal involvement), which was identified with linear strandings in the perigastric fat, they were regarded as locally advanced. However, organ invasion, peritoneal involvement, and pathological paraaortocaval LNs were also mentioned in the radiology report for clinical evaluation.

### 2.3 Restaging, Treatment Modifications, and Surgical Planning

Restaging was conducted after the administration of four cycles of chemotherapy and included CT and endoscopy when clinically warranted. This approach was designed to detect disease progression or the emergence of distant metastases. In the FLOT x8 group, patients underwent regular assessments after the fourth cycle to determine their eligibility to continue the remaining four cycles. In the FLOT x8 group, the following criteria were set for post‐four cycles interval radical surgery: (1) Intolerance to the FLOT chemotherapy regimen; (2) manifestation of unacceptable chemotherapy‐induced toxicity; (3) according to RECIST criteria [8], radiological evidence of disease progression post‐four cycles as determined by CT, yet retaining resectability, or the absence of a partial response or stable disease state.

The protocol in the event of intolerance during FLOT therapy for both groups consisted of dose adjustments made for specific drugs by the medical oncology team for specific toxicities or intolerances. In patients with febrile neutropenia, thrombocytopenia leading to bleeding, or any hematologic dose‐limiting toxicity despite prophylactic granulocyte‐colony stimulating factor use, the dose of chemotherapy agents was reduced by 25% and by 50% if these toxicities recurred after the initial dose reduction. A similar approach was applied for non‐hematologic dose‐limiting toxicities. Modification or cessation of the treatment is contemplated in the event of intolerable toxicity, disease progression, mortality, or patient request. Radical surgery was scheduled 3 weeks after completing all intended chemotherapy cycles for both groups.

### 2.4 Surgical Procedures and Pathological Assessment

Surgical procedures were determined by the anatomical location and extent of the primary tumor. Depending on these factors, either total or subtotal distal gastrectomy with D2 lymphadenectomy was performed, representing the standard of care at our institution. Pathological reports, compiled by expert pathologists, followed international recommendations and guidelines from the 8th edition of the TNM classification. Reports included morphological subtypes based on the WHO Classification of Tumors, tumor regression grade (TRG), tumor size, post‐neoadjuvant therapy differentiation grade, margin status, presence of lymphovascular and perineural invasion, number of harvested lymph nodes, and lymph node ratio calculated as the ratio of positive to total harvested LNs. Tumor response to neoadjuvant chemotherapy was quantified using the College of American Pathologists (CAP) scoring system [9].

### 2.5 Data Collection and Assessment

Data were collected prospectively and analyzed retrospectively, encompassing a range of parameters. Each patient follow‐up update is supported by institutional electronic records and the national personal health system, e‐Nabiz, constructed and validated by the Turkish Ministry of Health (https://enabiz.gov.tr/). The e‐Nabiz system securely stores personal health data in an encrypted format in compliance with data protection regulations. After obtaining the patient's consent, this data is only shared with healthcare providers. The demographic data gathered included age, sex, body mass index (BMI), American Society of Anesthesiologists (ASA) score, baseline Eastern Cooperative Oncology Group (ECOG) performance status, and patients' comorbidities.

The recorded clinical data covers the clinical stage at diagnosis, and the number of FLOT chemotherapy cycles each patient received as well as both non‐hematologic and hematologic toxicity that followed the chemotherapy. Perioperative outcomes were documented, including the surgical technique (open, laparoscopic, robotic, total, or subtotal gastrectomy), duration of surgery, and length of hospital stay. Postoperative complications, including anastomotic leaks, were also noted, and categorized according to the Clavien−Dindo classification. The pathological outcomes derived from pathology reports entail the pathological stage (T and N categories), the response to neoadjuvant chemotherapy as per CAP, and the detection of lymphatic, vascular, and perineural invasion.

Lastly, the follow‐up data focused on oncological outcomes. This included documentation of recurrence, disease‐free survival (DFS), and overall survival (OS) rates. The primary outcome was the completion rate of all intended chemotherapy cycles. Secondary outcomes included histopathological response, toxicity, and DFS and OS rates.

### 2.6 Statistical Analysis

Statistical analysis was performed using the SPSS version 28.0 (IBM Corp., Armonk, NY, USA). Continuous data were presented in mean ± standard deviation (SD), while categorical data were presented in number and frequency. The Mann−Whitney *U* test was used to analyze continuous data, while the chi‐square test was used to analyze categorical data. The Kaplan−Meier method was used for survival analysis, and the survival curves were compared using the log‐rank test. A *p*‐value of < 0.05 was considered statistically significant.

Propensity score matching with the nearest neighbor matching method (PSM) was used to minimize selection bias. The FLOT 4+4 and FLOT x8 groups were matched at a 1:1 ratio with a match tolerance of 0.2. Potential confounders that were considered clinically relevant variables, such as age, BMI, clinical stage at diagnosis, clinical stage, tumor location, and histology, were included in the propensity score model. After PSM, 37 patients remained in each group and were compared in terms of primary and secondary outcomes.

## 3 Results

The initial cohort of our study, before PSM, consisted of patients divided into two groups based on their FLOT chemotherapy regimen: 37 patients in the FLOT 4+4 group and 74 in the FLOT x8 group. The median age of this cohort was 63 years (range: 55−70), and the median BMI was recorded at 25.24 (range: 23−28.3). An analysis of this initial cohort revealed no significant differences in key demographic and clinical characteristics, including age, BMI, ASA score, baseline ECOG performance status (which was 0 or 1 in both groups), Lauren classification, clinical stage, and tumor location. The surgical procedures performed, including total and subtotal gastrectomy and techniques such as open, laparoscopic, and robotic surgeries, were similarly distributed among the groups. Following PSM, our study consisted of 37 patients in each treatment group, with a mean age of 64 years (range: 55−70). There were no significant differences between the two groups in terms of age, BMI, gender distribution, ASA score, and tumor location. The age distribution was 62 (37−83) in the FLOT 4+4 group and 64 (42−77) in the FLOT x8 group, while BMI ranged from 25.24 (18.1−33.2) to 26.5 (18.1−34.3), respectively. Patient demographics and characteristics, such as gender distribution, ASA scores, and tumor locations (fundus and corpus vs. corpus, antrum, and pylor), were comparable between the two groups (Table 1).

**Table 1:** Patient and tumor characteristics before and after propensity score matching.

	Before PSM^a^			After PSM		
	FLOT 4+4	FLOT x8	*p* value	FLOT 4+4	FLOT x8	*p* value
	(*n* = 37)	(*n* = 74)		(*n* = 37)	(*n* = 37)	
Age	62 [49.5−70]	63 [55.75−71]	0.42	62 [37−83]	64 [42−77]	0.27
BMI	25.25 [23.5−27.6]	25.3 [22.9−28.48]	0.9	25.24 [18.1−33.2]	26.5 [18.1−34.3]	0.7
Gender			0.57			1
Female	12 (32.4%)	28 (37.8%)		12 (32.4%)	12 (32.4%)	
Male	25 (67.6%)	46 (62.2%)		25 (67.6%)	25 (67.6%)	
Lauren classification			0.68			0.24
Diffuse	19 (51.4%)	35 (47.3)		18 (48.6%)	13 (35.1%)	
Intestinal	18 (48.6%)	39 (52.8%)		19 (51.4%)	24 (64.9%)	
Clinical stage			0.82			0.15
I	2 (5.4%)	6 (8.1%)		2 (5.4%)	4 (10.8%)	
II	11 (29.7%)	19 (25.7%)		11 (29.7%)	16 (43.2%)	
III	24 (64.9%)	49 (66.2%)		24 (64.9%)	17 (46%)	
ASA			0.57			0.61
I	3 (8.1%)	6 (8.1%)		3 (8.1%)	3 (8.1%)	
II	22 (59.5%)	45 (60.8%)		22 (59.5%)	19 (51.4%)	
III	11 (29.7%)	23 (31.1%)		11 (29.7%)	15 (40.5)	
IV	1 (2.7%)	0		1	0	
Tumor location			0.65			0.78
Proximal (cardia and fundus)	9 (24.3%)	21 (28.4%)		9 (24.3%)	8 (21.6%)	
Middle or distal (corpus, antrum and pylor)	18 (75.7%)	53 (71.6%)		28 (75.7%)	29 (78.4%)	

^a^ Propensity score‐matching.

In terms of primary endpoint, the all‐cycle completion rate of FLOT treatment was significantly higher in the FLOT x8 (89.1%) compared to the 4‐cycle group (67.6%) (*p* < 0.001) (Table 2). In the FLOT 4+4 group, one patient could not complete preoperative treatment due to toxicity, and 7 (18.9%) patients could not start postoperative chemotherapy due to postoperative complications (*n* = 5) and intolerance (*n* = 2). In the FLOT x8 group, all patients completed the initial four cycles of FLOT therapy, avoiding the need for interval surgery and proceeding with the remaining cycles. However, three patients were unable to complete the full eight cycles: two received five cycles, and one received six cycles. All patients in this group proceeded to surgery after TNT. The cumulative dose of chemotherapy cycles in the FLOT 4+4 group was 255 out of 296 cycles, while it was 288 out of 296 cycles in the FLOT x8 group. Peri‐ and postoperative outcomes, including duration of surgery, length of hospital stay, anastomotic leaks, and complications as classified by the Clavien−Dindo grading system, showed no significant differences between the two groups (Table 2).

**Table 2:** Perioperative, postoperative outcomes, and histopathological results after propensity score matching.

	FLOT 4+4	FLOT x8	*p* value
	(*n* = 37)	(*n* = 37)	
Resection type			1
Total	34 (91.9%)	34 (91.9%)	
Subtotal	3 (8.1%)	3 (8.1%)	
Surgical technique			0.06
Open	10 (27%)	19 (51.4%)	
Minimally invasive technique	27 (73%)	18 (48.6%)	
Laparoscopic	19 (51.4%)	4 (10.8%)	
Robotic	8 (21.6%)	14 (37.8%)	
Duration of surgery	195 [120−370]	200 [120−425]	0.85
Length of hospital stay	8 [5−45]	9 [5−25]	0.43
FLOT completion rate	25 (67.6%)	33 (89.1%)	< 0.01
Hematologic toxicity			0.3
Grade 1	6 (28.6%)	9 (40.9%)	
Grade 2	9 (42.9%)	5 (22.7%)	
Grade 3	5 (23.8%)	4 (18.2%)	
Grade 4	1 (4.8%)	4 (18.2%)	
Non‐hematologic toxicity			0.07
No toxicity	12 (54.5%)	13 (61.9%)	
Grade 1	0	2 (9.5%)	
Grade 2	7 (31.8%)	3 (14.3%)	
Grade 3	3 (13.6%)	3 (14.3%)	
Grade 4	0	0	
Need for G‐CSF use	15 (68.2%)	15 (71.4%)	0.82
Febrile neutropenia	0	1 (4.8%)	0.98
Number of harvested lymph nodes	42.5 ± 15.7	41.2 ± 17.25	0.73
Lymph node positivity rate	0.098 ± 0.16	0.081 ± 0.21	0.24
Anastomotic leak	4 (10.8%)	3 (8.1%)	0.69
Clavien−Dindo scores of postoperative complications			0.46
0	8 (21.6%)	8 (21.6%)	
1	8 (21.6%)	10 (27%)	
2	12 (32.4%)	8 (21.6%)	
3	5 (13.5%)	10 (27%)	
4	3 (8.1%)	1 (2.7%)	
5	1 (2.7%)	0	
Pathologic stage			0.48
0	3 (8.1%)	7 (18.9%)	
1	11 (29.7%)	11 (29.7%)	
2	6 (16.2%)	6 (16.2%)	
3	17 (45.9%)	12 (32.4%)	
4	0	1 (2.7%)	
CAP score			0.25
Grade 0	3 (8.1%)	7 (18.9%)	
Grade 1	11 (29.7%)	6 (16.2%)	
Grade 2	12 (32.4%)	16 (43.2%)	
Grade 3	11 (29.7%)	8 (21.6%)	
Complete response	3 (8.1%)	7 (18.9%)	0.18
Lymphatic invasion	13 (36.1%)	13 (36.1%)	0.93
Vascular invasion	7 (19.4%)	6 (16.6%)	0.56
Perineural invasion	12 (33.3%)	10 (27.8%)	0.48

Abbreviations: CAP score, College of American Pathologists score; G‐CSF, granulocyte colony‐stimulating factor.

**Figure 2:**
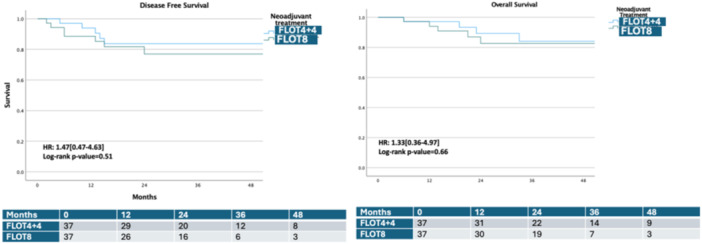
Kaplan−Meier estimates of disease‐free survival and overall survival for the FLOT 4+4 and FLOT x8 groups.

**Figure 3:**
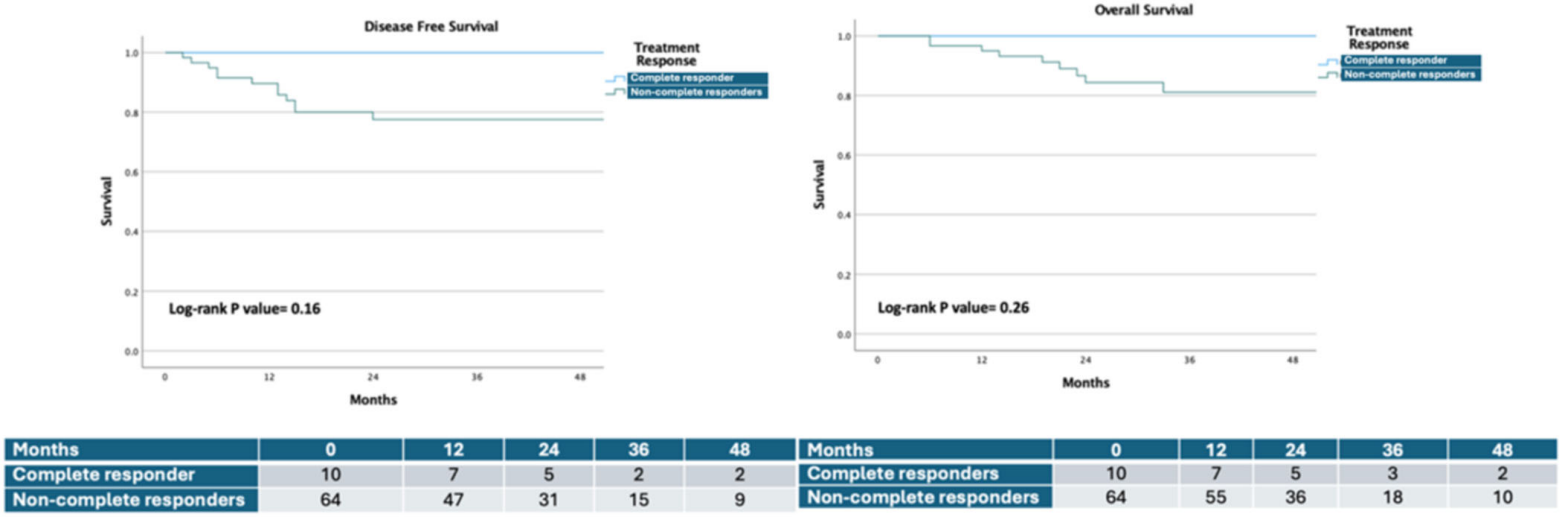
Kaplan−Meier estimates of disease‐free survival and overall survival for the complete versus non‐complete responders.

Regarding secondary endpoints, there were no significant differences between the groups regarding hematologic and non‐hematologic toxicity (*p *= 0.11 and *p* = 0.07, respectively) (Table 2). Analysis of the CAP score revealed a trend toward a higher complete response rate in the FLOT x8 group (18.9%) compared to the FLOT 4+4 group (8.1%), though this did not reach statistical significance (*p *= 0.25). R0 resection rate was 100% in both groups. The mean number of harvested lymph nodes was 42.5 ± 15.7 and 41.2 ± 17.25 in FLOT 4+4 and FLOT x8 groups, respectively (*p *= 0.73). Similar rates of lymph node positivity were identified (0.098 ± 0.16 in the FLOT 4+4 group and 0.081 ± 0.21 in the FLOT x8 group [*p *= 0.24]). At the median follow‐up of 36 months, DFS and OS rates also showed no significant differences, with DFS rates at 83.7% for the FLOT 4+4 group and 76.9.3% for the FLOT x8 group (*p *= 0.51, HR: 1.47 [0.47−4.63]) and OS rates of 84.1% and 82.6%, respectively (*p *= 0.66, HR: 1.33 [0.36−4.97]) (Figure 2). At the same follow‐up, all complete responders (*n* = 10) were disease‐free and alive (Figure 3).

## 4 Discussion

The primary rationale for adopting the FLOT x8 regimen lies in its potential to optimize chemotherapy adherence and completion rates, which is a critical issue highlighted by the low postoperative completion rates in both the MAGIC and FLOT 4‐AIO studies [2, 3]. Our study demonstrated that administering all FLOT cycles preoperatively, in the form of TNT, can address the challenge of treatment adherence, achieving an all‐cycle completion rate of 89.1%. The cumulative dose of FLOT cycles administered was higher with the TNT approach compared to the perioperative regimen. Although the complication rates were similar between the two groups, 19% of patients in the FLOT 4+4 group were unable to initiate postoperative chemotherapy. This finding suggests that these patients might have benefited from a TNT approach, as it would have allowed them to complete more chemotherapy cycles preoperatively, potentially improving their overall outcomes.

Another notable finding was that patients tolerated TNT well with the FLOT regimen, with comparable rates of toxicity documented in the perioperative FLOT regimen. The toxicity rates in our cohort were comparable to the serious adverse events rate of 27% reported in the study conducted by Al‐Batran et al. [3]. These adverse events were associated with the treatment, including those that occurred while patients were hospitalized for surgery. In addition to that finding, TNT for LARG and gastroesophageal cancer did not result in any notable delay in surgery, prolonged hospital length of stay, or increased surgical morbidity, demonstrating its feasibility and safety. The 30% Clavien−Dindo grade III−IV postoperative complication rate observed in our TNT cohort is favorable compared to previously reported rates for standard perioperative regimens [2, 3, 10, 11, 12]. Our findings align with those reported in a recent retrospective analysis by Ganschow et al., which found no significant increase in perioperative complications with intensified neoadjuvant chemotherapy consisting of six cycles of FLOT regimen compared to standard perioperative chemotherapy or upfront surgery [13].

A recently published retrospective cohort study conducted by Yang et al. LARGC patients undergoing TNT or perioperative chemotherapy at Memorial Sloan Kettering Cancer Center analyzed 121 patients who received perioperative chemotherapy and 28 patients treated with TNT with some degree of heterogeneity in chemotherapy regimen (79% of the TNT cohort received FLOT, and the remaining received other regimens) [14]. Despite the higher proportion of patients in TNT patients achieving pathologic complete response (14% vs. 5.8%), no significant differences were identified in recurrence‐free and OS rates in the TNT and perioperative groups at 24 months. Aligning with these findings, delivering more cycles of the FLOT regimen as TNT resulted in a higher, though not statistically significant, pathological complete response rate in our cohort. Although these improved histopathological outcomes did not translate into a survival benefit in our study, we acknowledge from pivotal studies in various malignancies that achieving a pathological complete response after neoadjuvant treatment predicts better survival by indicating the absence of residual cancer cells, thereby reducing the risk of recurrence and improving prognosis [15, 16, 17]. Further studies with larger cohorts and extended follow‐up periods are necessary to validate the survival benefits conferred by achieving pathological complete response and to identify the subsets of patients most likely to benefit from the FLOT x8 regimen.

In our cohort, all pathologically complete responders in both groups were disease‐free and alive within 36 months after surgery. This outcome resembles the revolutionary advances in multimodal treatment that eventuates TNT in rectal cancer treatment [18, 19]. Similar to the journey in rectal cancer treatment, future treatment paradigms of gastric cancer patients would include nonoperative management of carefully selected subgroups, meeting the criteria of complete clinical response based on modern imaging, and endoscopic evaluation modalities after TNT.

Innovative trial designs of the TNT approach incorporating novel systemic therapies, including targeted therapies and immunotherapies, would optimize the future directions of LARGC. The integration of immunotherapy into the treatment paradigm for resectable gastric cancer represents a significant evolution in clinical practice. Recent trials, such as the GASPAR, MATTERHORN, DANTE/IKF‐s633, and KEYNOTE‐585 studies, have explored the combination of immune checkpoint inhibitors like spartalizumab, durvalumab, atezolizumab, and pembrolizumab, respectively, with the FLOT regimen, offering promising, albeit preliminary, results [20, 21, 22, 23]. Although the addition of immune checkpoint inhibitors to the FLOT regimen has shown significant improvements in pathological complete response in all these trials, it is not yet a standard approach because only the KEYNOTE‐585 trial reported event‐free survival (EFS), with no significant contribution with addition of immunotherapy, and other trial results of EFS outcomes were not yet available. These outcomes suggest that while the FLOT regimen may remain a vital component of perioperative treatment, its role may evolve as more data becomes available. Future research should focus on identifying specific patient populations that would benefit most from immunotherapy, optimizing treatment sequencing, and exploring the potential for immunotherapy to either complement or, in some cases, replace traditional chemotherapy regimens like FLOT. Until more definitive evidence emerges, the FLOT regimen, particularly in combination with immunotherapy, continues to be a cornerstone in the multimodal treatment of resectable gastric cancer, with ongoing trials likely to shape its future application.

The integration of artificial intelligence (AI) and circulating tumor DNA (ctDNA) into the treatment landscape of gastric cancer may represent a significant advancement. AI can enhance the precision of treatment plans by predicting patient responses to chemotherapy and identifying those who are most likely to benefit from TNT, thereby personalizing treatment and potentially improving outcomes. [24] Additionally, ctDNA analysis for gastric cancer offers a noninvasive method to monitor tumor dynamics, refine patient selection, detect minimal residual disease, and assess treatment response in real‐time. [25] These technologies could enable earlier intervention and adjustment of treatment strategies, ultimately aiming to improve survival rates and reduce recurrence.

The study is limited by the relatively small sample size in the groups and the bias inherent to a retrospective analysis concerning patient selection, and the relatively short follow‐up. Also, because these treatment approaches are ever evolving, we started employing TNT only in 2020, and thus, the survival information is relatively short. At the same time, these preliminary results are striking both in terms of the safety of TNT as well as its potential efficacy by allowing completion of the full protocol in about 90% of the cases. In contrast, the standard protocol has to be discontinued in about a third of the cases. This study also highlights the paramount importance of comparing perioperative and TNT results for LARGC managed by the same multidisciplinary team with significant experience in gastrointestinal oncology. Long‐term outcomes of TNT with FLOT regimen followed by gastrectomy with standardized D2 lymphadenectomy are essential.

In summary, the FLOT regimen's TNT corresponds with higher treatment completion rates, a similar safety profile to the standard perioperative scheme, and a twofold increase in pathological complete response rate. While there was no significant difference in DFS and OS between the FLOT 4+4 and TNT groups, all complete responders were disease‐free and alive, irrespective of the treatment scheme. This study supports the need for future research to evaluate whether TNT with the FLOT regimen offers oncological advantages and to identify the specific group of patients who may benefit most.

## Author Contributions

Ahmet Rencuzogullari, Emre Balik, Sezer Saglam, and Dursun Bugra participated in the concept and study design. Ahmet Rencuzogullari, Salih Nafiz Karahan, Sahin Lacin, Orhun Cig Taskin, Burcu Saka, Duygu Karahacioglu, Bengi Gurses, Ibrahim Halil Ozata, Emre Ozoran, and Derya Salim Uymaz participated in patient recruitment, data acquisition, and manuscript drafting. Ahmet Rencuzogullari and Salih Nafiz Karahan are involved in manuscript writing. Dursun Bugra, Emre Balik, Sezer Saglam, and Fatih Selcukbiricik are involved in drafting the work or revising it critically for important intellectual content and final approval of the version to be published. Ahmet Rencuzogullari, Dursun Bugra, and Emre Balik were involved in supervision, final writing, and editing.

## Ethics Statement

The Ethics Committee of the Koç University approved this retrospective study (2022.439.IRB1.165).

## Conflicts of Interest

The authors declare no conflicts of interest.

## Synopsis

This retrospective study compares total neoadjuvant FLOT therapy (FLOT x8) with standard‐of‐care perioperative therapy (FLOT 4+4) for locally advanced resectable gastric cancer. Total neoadjuvant therapy with the FLOT x8 regimen results in higher chemotherapy completion rates and a higher, though not statistically significant, pathological complete response rate, with comparable toxicity and survival outcomes to the FLOT 4+4 regimen.

## Data Availability

The data that support the findings of this study are available from the corresponding author upon reasonable request.
